# Highly radiative emission of room temperature–localized excitons enabled by charge-neutralized 0D quantum wells in 2D semiconductors

**DOI:** 10.1126/sciadv.ady2186

**Published:** 2026-03-13

**Authors:** Taeyoung Moon, Hyeongwoo Lee, Jihae Lee, Dong Kyo Oh, Soo Ho Choi, Yeonjeong Koo, Christopher E. Stevens, Hyunje Cho, Deep Jariwala, Je-Hyung Kim, Moon-Ho Jo, Joshua R. Hendrickson, Ki Kang Kim, Junsuk Rho, Yung Doug Suh, Kyoung-Duck Park

**Affiliations:** ^1^ Department of Physics, Pohang University of Science and Technology (POSTECH), Pohang 37673, Republic of Korea.; ^2^ Center for Multidimensional Carbon Materials (CMCM), Institute for Basic Science (IBS), Ulsan 44919, Republic of Korea.; ^3^ Department of Chemical Engineering, Pohang University of Science and Technology (POSTECH), Pohang 37673, Republic of Korea.; ^4^ Department of Mechanical Engineering, Pohang University of Science and Technology (POSTECH), Pohang 37673, Republic of Korea.; ^5^ Department of Electrical and Computer Engineering, Nick Holonyak, Jr. Micro and Nanotechnology Laboratory, University of Illinois Urbana-Champaign, Urbana, IL 61801, USA.; ^6^ Air Force Research Laboratory, Sensors Directorate, Wright-Patterson Air Force Base, Dayton, OH 45433, USA.; ^7^ Center for Van der Waals Quantum Solids, Institute for Basic Science (IBS), Pohang 37673, Republic of Korea.; ^8^ Department of Materials Science and Engineering, Pohang University of Science and Technology (POSTECH), Pohang 37673, Republic of Korea.; ^9^ Department of Electrical and Systems Engineering, University of Pennsylvania, Philadelphia, PA 19104, USA.; ^10^ Department of Physics, Ulsan National Institute of Science and Technology, Ulsan, Republic of Korea.; ^11^ Department of Energy Science, Sungkyunkwan University (SKKU), Suwon 16419, Republic of Korea.; ^12^ Department of Electrical Engineering, Pohang University of Science and Technology (POSTECH), Pohang 37673, Republic of Korea.; ^13^ POSCO-POSTECH-RIST Convergence Research Center for Flat Optics and Metaphotonics, Pohang 37673, Republic of Korea.; ^14^ National Institute of Nanomaterials Technology (NINT), Pohang 37673, Republic of Korea.; ^15^ Department of Chemistry & Graduate School of Semiconductor Materials and Devices Engineering, Ulsan National Institute of Science and Technology (UNIST), Ulsan 44919, Republic of Korea.; ^16^ Department of Semiconductor Engineering, Pohang University of Science and Technology (POSTECH), Pohang 37673, Republic of Korea.; ^17^ Institute for Convergence Research and Education in Advanced Technology, Yonsei University, Seoul 03722, Republic of Korea.

## Abstract

Nondiffusing localized excitons (X_L_) in two-dimensional semiconductors present a robust platform for mediating light-matter interactions, with potential applications in both photovoltaics and light-emitting devices. However, at room temperature, high thermal energy hinders X_L_ formation, while excess charges diminish the quantum yield (QY) through nonradiative decay. Here, we present high-QY X_L_ emission in ambient conditions by removing excess charges and inducing efficient exciton funneling into a Au nanohole. Specifically, by evaporating an H_2_O barrier between the n-type MoS_2_ and the Au substrate, we induce a grounding effect on electrons. Dominantly populating excitons are then funneled and bound to the nanohole through the strain-induced zero-dimensional quantum well effect. We confirm the exciton confinement efficiency of ~98% using a drift-diffusion model, enabling bright X_L_ emission at the nanoscale. Using tip-induced gigapascal-scale pressure, we control X_L_ dynamics and QY in a reversible manner. Our approach provides an innovative strategy for X_L_-based nanophotonic devices.

## Teaser

Charge-neutralized quantum wells enable highly efficient light emission from localized excitons at room temperature.

## INTRODUCTION

Neutral excitons (X_0_) in two-dimensional (2D) semiconductors are of great interest for mediating light-matter interactions, with diverse applications in optoelectronic devices (*1*–*3*). However, the mobile nature of X_0_, such as diffusion and drift, poses challenges in commercializing stable excitonic devices, particularly in 2D materials compared to excitonic 0D and 1D materials (*4*–*6*). In contrast, localized excitons (X_L_) offer a distinct advantage as robust mediators of light-matter interactions due to their stable confinement at localized positions (*7*–*15*). Beyond the recently demonstrated single-photon emission properties (*11*–*20*), X_L_ also holds potential as a mediating quasiparticle for light-matter interactions, with promising applications in fields, such as photovoltaics (*21*) and light-emitting devices (*22*–*24*). However, at room temperature, the formation of X_L_ is difficult, as the dominant population of X_0_ is driven by the high thermal energy of the environment. This thermal energy increases exciton-phonon scattering and kinetic energy, leading to delocalization of excitons.

While X_L_ can form in 2D semiconductors (*7*–*15*, *25*–*27*), their quantum yield (QY) remains low, primarily because of excess charges within the crystal (*5*, *28*, *29*), which act as nonradiative decay pathways and favor the formation of trions (X–) (*30*–*32*). As a result, naturally grown or transferred 2D semiconductors struggle to achieve bright X_L_ emission at room temperature.

To address the challenge of diffusive exciton transport and confine excitons within a local potential, strain-gradient engineering approaches have recently been explored at both the microscale (*30*, *33*–*35*) and nanoscale areas (*11*–*18*). These advancements in nanoscale strain–gradient engineering have notably enhanced exciton funneling efficiency, opening the door to effective generation of X_L_, yet ideal platform for confined X_L_ at the nanoscale has not been investigated.

To further overcome the issue of reduced QY due to excess charges, electrostatic doping through gate voltage modulation has been proposed (*11*, *36*). By reducing excess electrons in n-type MoS_2_ monolayers (MLs), nonradiative decay pathways of excitons can be suppressed, resulting in near-unity emission QY (*2*, *37*, *38*). However, the complexity of fabricating electrical devices and scalability challenges have hindered practical applications. Thus, achieving robust X_L_ emission at room temperature with high-QY at spatially deterministic positions, using a simple and easily implementable approach, remains highly desirable.

In this work, we demonstrate robust and high-QY X_L_ emission under ambient conditions by eliminating excess charges and driving efficient exciton funneling into a Au nanohole. By evaporating a residual H_2_O layer between the n-type MoS_2_ and the Au substrate around the nanohole, we induce a grounding effect that effectively neutralizes net charges of the crystal. This interfacial H_2_O layer naturally forms during the transfer process under ambient conditions and is known to degrade the performance of transition metal dichalcogenides (TMD) devices by hindering charge transfer (*39*, *40*). As a result, excitons are funneled and confined within the nanohole via a strain-induced quantum well effect. Using a drift-diffusion model, we quantify an exciton confinement efficiency of ~98%, leading to highly stable and bright X_L_ emission precisely at targeted nanoscale regions. Furthermore, by applying tip-induced gigapascal-scale pressure, we achieve reversible control over X_L_ dynamics and emission QY. This innovative approach opens different avenues for X_L_-based nanophotonic devices, offering a promising solution that has, until now, been largely overlooked.

## RESULTS

### A nanoscale excitonic emitter with high populations of X_L_

**Fig. 1.**
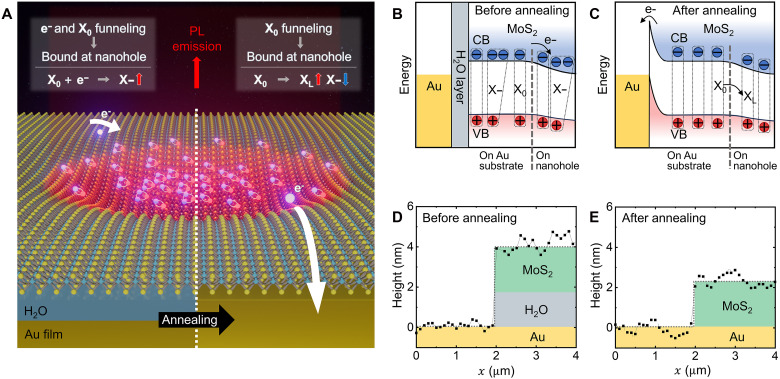
Schematic illustrations of the experimental design for dominant radiative decay of X_L_ at the nanohole. (**A**) Illustration of effectively funneled and bound excitons at the nanohole, with dominant radiative decay, facilitated by the electron quenching process in the strained-MoS_2_ ML. Energy band diagrams of the strain-induced 2D crystal before (**B**) and after (**C**) thermal annealing. AFM height profiles of the MoS_2_ ML on the Au film before (**D**) and after (**E**) thermal annealing, confirming the removal of the H_2_O layer.

To induce the bound excitons at the localized area and enhance the radiative decay rate of them, we fabricate nanohole structures to induce strain-gradient in a MoS_2_ ML and perform thermal treatment to suppress nonradiative decay pathways caused by excess electrons, as illustrated in Fig. 1A. Specifically, we dry-transfer a MoS_2_ ML onto a 500-nm diameter nanohole to induce tensile strain on the crystal (see Materials and Methods for the details). In this case, nanoscale strain gradient leads to exciton funneling with much higher efficiency than that of microscale strain gradient, as demonstrated previously (*32*). However, for the n-type MoS_2_, both electrons and excitons funnel together into the nanohole, resulting in a reduction in exciton population due to the exciton-to-trion conversion and low QY for the remaining excitons due to the nonradiative decay into the excess electrons. By removing the H_2_O layer between the MoS_2_ and the Au film through thermal annealing at 300°C for an hour under vacuum conditions (~8 mTorr) (*41*–*44*), we can extract electrons of the MoS_2_ ML into the Au film, as shown in Fig. 1 (B and C). Before thermal annealing, an interfacial layer composed predominantly of H_2_O, which forms during the polydimethylsiloxane (PDMS)–based dry transfer process under ambient conditions, acts as a dielectric barrier that blocks electron movement. Consequently, electrons funnel into the nanohole and contribute to trion formation (Fig. 1B). In contrast, after thermal annealing, the free electrons in the MoS_2_ flow into the Au film due to the work function difference between them, allowed by removal of the dielectric barrier (Fig. 1C) (*45*, *46*). Consequently, we achieve high-QY photoluminescence (PL) emission of X_L_ at the deterministic nanoscale area. We confirm the removal of the H_2_O layer through atomic force microscopy (AFM) height profiles of transferred MoS_2_ before (Fig. 1D) and after (Fig. 1E) thermal annealing. The decreased height of ~2 nm is attributed to the removal of H_2_O layer, which naturally exists in the ambient conditions. Note that the measured height of ~2 nm for the transferred MoS_2_ is higher than that of pristine TMD (~0.7 nm), indicating the presence of residual contaminants (*39*, *47*, *48*).

### Effects of H_2_O layer on funneling and emission properties for the MoS_2_ ML on 1D nanogap

**Fig. 2.**
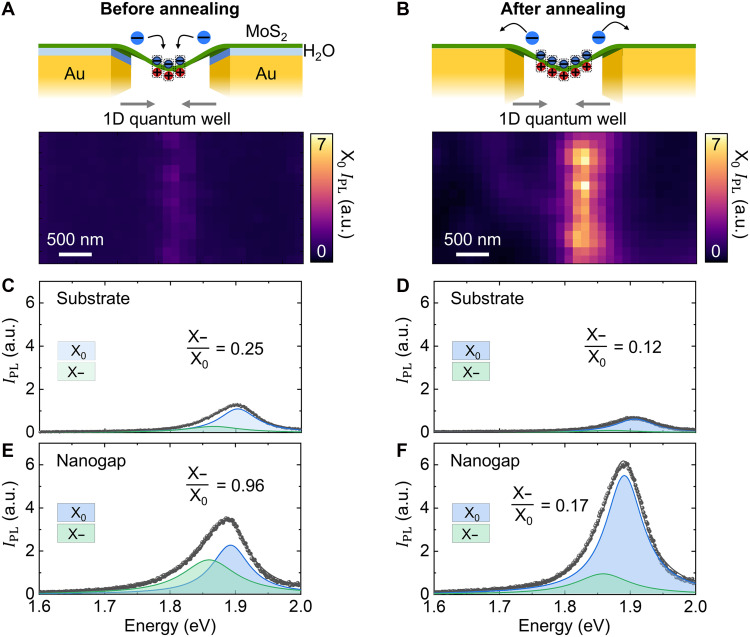
Hyperspectral PL imaging of the strained MoS_2_ ML at the 1D nanogap. X_0_ PL peak intensity (*I*_PL_) images of the strained MoS_2_ on the 1D nanogap before (**A**) and after (**B**) thermal annealing, illustrating funneling dynamics of excitons and electrons under the 1D potential confinement. PL spectra of the MoS_2_ ML on a Au substrate before (**C**) and after (**D**) thermal annealing. PL spectra of the strained MoS_2_ ML at the Au nanogap before (**E**) and after (**F**) thermal annealing. The PL spectra are fitted to a Lorentzian function, where the black line represents the fit to the raw spectra, while the blue and green lines correspond to the fits for the neutral exciton (X_0_) and trion (X−). The black dots indicate the raw data points. a.u., arbitrary units.

To investigate the effect of the nanoscale strain gradient and the removal of dielectric H_2_O layer systematically, we first characterize the PL properties of the MoS_2_ ML transferred on the ~100-nm width 1D Au nanogap, before and after thermal annealing. Figure 2A shows the X_0_ PL intensity image of the strained MoS_2_ ML on the Au nanogap before thermal annealing, resulted from funneling of excitons and electrons to the lower energy state (*17*, *30*–*32*, *35*). After thermal annealing, the electrons in the MoS_2_ ML are extracted into the Au film, restricting electron funneling toward strained MoS_2_ ML (*45*, *46*), leading to a subsequent decrease of trion density and Auger recombination at the nanogap region. This electron quenching leads to high-QY emission for the funneled excitons of the strained MoS_2_ ML at the nanogap (*2*, *37*, *38*), as shown in Fig. 2B. We clearly observe the PL intensity change with spectral modifications at the Au substrate and nanogap regions before and after the thermal annealing in Fig. 2 (C to F). On the Au substrate region, both the X_0_ and X− PL intensities decrease with the reduced X−/X_0_ PL intensity ratio after thermal annealing (Fig. 2, C and D). This result is attributed to the electron quenching on the Au substrate, which restricts the high populations of X_0_ and conversion into X−. On the nanogap region, we can clearly see the enhanced PL of both X_0_ and X− compared to the PL properties at the Au substrate region (Fig. 2, E and F). Before thermal annealing, both excitons and electrons funnel into the strained region of crystal, which leads to enhanced PL of X_0_, as well as X−, through the exciton-to-trion conversion. After thermal annealing, the electron quenching effect is clearly observed with decreased X− PL intensity and highly increased X_0_ PL intensity. In addition to the reduced X− conversion, PL QY of X_0_ is highly increased because of the suppressed nonradiative Auger recombination process, which is induced by the extra charges. Therefore, through the 1D nanogap structure with the thermal annealing process removing the H_2_O layer, we can achieve high-QY excitonic emitter at the desired spatial position. Note that the slight spectral red shift is attributed to the tensile strain effect at the nanogap region.

### Robust X_L_ emission at room temperature from the strained MoS_2_ ML on 0D nanohole

We then transfer the MoS_2_ ML onto the nanohole with a 500-nm diameter to induce and probe the 0D confinement effects of excitons. In this 0D potential well, a higher degree of exciton confinement, or more efficient exciton funneling, is anticipated compared to 1D potential wells (*49*). This is because the 2D strain gradient structure creates deeper potential wells (*30*). In addition, in this configuration, the exciton binding energy (*E*_b_) at the nanohole is notably enhanced because of the increased Coulomb interaction between excitons in the tightly confined 0D space, which corresponds to the lowest bandgap region at the nanohole center (*9*, *10*). These mechanisms enable the formation of robust X_L_ at the nanohole, even at room temperature (*14*, *16*). However, as observed in the case of 1D nanogaps (Fig. 2, E and F), excess electrons at the potential well lead to trion formation and increase the nonradiative decay rate. To mitigate this, thermal annealing is critical for removing the H_2_O layer, facilitating robust X_L_ emission at the nanohole under ambient conditions.

**Fig. 3.**
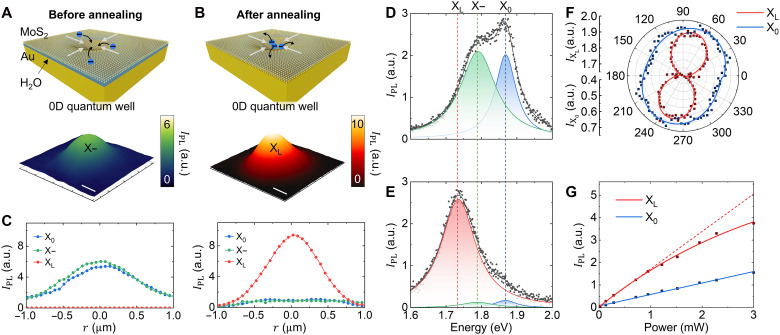
Robust X_L_ emission at room temperature via dominant radiative decay of effectively funneled excitons bound at the nanohole. (**A**) X− PL intensity image of the strained MoS_2_ ML at the nanohole before thermal annealing, with an illustration of electron funneling into the 0D potential well. (**B**) X_L_ PL intensity image of the strained MoS_2_ ML at the nanohole after thermal annealing, with an illustration of electron quenching into the Au substrate. Scale bar, 500 nm. (**C**) Line profiles of X_0_, X−, and X_L_ PL intensities before (left) and after (right) thermal annealing, derived from the center line of the nanohole. Corresponding PL spectra of the strained MoS_2_ ML before (**D**) and after (**E**) thermal annealing, demonstrating the robust X_L_ emission after thermal annealing. (**F**) Polarization-dependent PL intensities of X_L_ and X_0_, exhibiting linearly polarized emission of X_L_. (**G**) Excitation power-dependent PL intensities of X_L_ and X_0_, showing a saturation behavior of X_L_.

Figure 3A shows the X− PL intensity image of the sample before thermal annealing. The line profiles of X_0_, X−, and X_L_ PL intensity (Fig. 3C, left), as well as the PL spectrum at the nanohole (Fig. 3D), reveal that X_0_ and X− populations dominate because of the influence of excess electrons. Figure S1 further confirms exciton-to-trion conversion through hyperspectral imaging of MoS_2_ ML at various nanoholes, revealing strain-induced X− conversion. In addition, the X_0_ and X− dynamics are quantified using the mass action equation in fig. S2, demonstrating that electron quenching suppresses trion formation. On the other hand, after thermal annealing, X_L_ emission becomes dominant at the nanohole, as seen in the decreased PL intensities of X_0_ and X− in Fig. 3, (B and C, right, and E). This strong X_L_ emission is facilitated because electron quenching into the Au substrate suppresses trion formation and nonradiative Auger recombination at the nanohole. Quantitative analysis using rate-equation models (section S12) reveals that thermal annealing dramatically enhances the PL QY from 0.076 to 10% in the nanohole region, representing a 130-fold improvement that notably exceeds typical values for pristine MoS_2_ MLs (<1%). This substantial enhancement confirms the high-QY nature of X_L_ emission achieved through our combined approach of charge neutralization and strain-induced confinement. The robust X_L_ emission observed at the nanohole is a distinct feature of 0D confinement compared to 1D confinement, benefiting from stronger spatial confinement and a synergistic effect of reduced excess charge density. The spectral feature of a red-shifted X_L_ emission peak (Fig. 3E) supports the presence of X_L_ states. Our nanohole structure achieves an X_L_/X_0_ PL intensity ratio of ~15, notably higher than typical strain-engineering approaches where X_L_ and X_0_ PL intensities remain nearly comparable (*15*, *16*). This dramatic enhancement results from efficient quenching of excitons to the Au substrate in nonsuspended regions, effectively suppressing the X_0_ background while preserving bright X_L_ emission within the strain-localized nanohole area. Figure S3 provides additional hyperspectral evidence of enhanced X_L_ confinement after thermal annealing. Cryogenic temperature measurements (5 K) further validate the localized excitonic nature of this emission (see fig. S4). In addition, polarization- and excitation power–dependent PL measurements provide further evidences. Figure 3F presents polarization-resolved PL measurements for the X_0_ and X_L_ peaks, showing linearly polarized X_L_ emission consistent with prior studies (*50*). However, unlike single-exciton systems (*50*), the origin of this polarization differs, as the observed dipolar emission arises from ensemble averaging over multiple strain-confined excitonic states. The polar intensity plot was corrected for polarization-dependent instrumental losses, i.e., in the beam splitter, to reveal intrinsic emission properties. Figure S5 further compares PL spectra measured along two orthogonal polarization directions, highlighting the linear polarization of X_L_ emission in strained MoS_2_. Across multiple nanoholes, we consistently observed negligible polarization dependence for X_0_, while X_L_ exhibited pronounced dipolar emission patterns (see fig. S6). Figure 3G shows power-dependent PL intensity curves for X_0_ and X_L_, with X_L_ exhibiting saturation behavior with increasing excitation power. This observation is consistent with previous studies on X_L_ in 2D semiconductors (*50*, *51*). The less pronounced saturation behavior originates from the multiple closely spaced excitonic sublevels in a strain-induced potential well formed by the 500-nm nanohole. At room temperature, these excitons can populate several states simultaneously, delaying saturation within our experimental power limits. In addition, enhanced phonon scattering activates additional nonradiative recombination channels, substantially increasing the excitation power needed to approach any PL saturation (*52*–*55*). This multisublevel nature highlights the potential for future optimizations. For instance, reducing the nanohole size to below 100 nm could confine excitons to a single state and enable high-efficiency single-photon emission at room temperature, which is highly desirable for quantum information technologies.

### Tip-induced dynamical modulation of exciton confinement and X_L_ emission

**Fig. 4.**
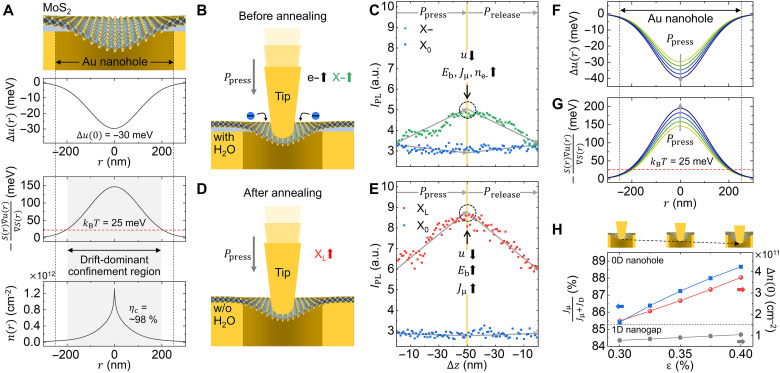
Simulated exciton funneling dynamics at the nanohole and experimental results of tip-induced modulation of exciton confinement and X_L_ emission. (**A**) Simulation results showing the bandgap-energy modification [Δ*u*(*r*), top], the drift-to-diffusion flow ratio of excitons [−*S*(*r*)∇*u*(*r*)/∇*S*(*r*), middle], and the exciton density [*n*(*r*), bottom] with as a function of the distance *r* from the center for the strain-induced MoS_2_ ML on the nanohole. Schematic illustration of tip-induced modulation of exciton behavior under tip-induced pressure (*P*_press_) at the nanohole is shown before (**B**) and after (**D**) thermal annealing. PL intensity of excitonic emissions (X–, X_0_, and X_L_) as a function of tip displacement (Δ*z*) before (**C**) and after (**E**) thermal annealing, attributed to modifications in *u*, *E*_b_, *J*_μ_, and number of electrons (*n*_e–_). Simulated spatial profiles of Δ*u*(*r*) (**F**) and −*S*(*r*)∇*u*(*r*)/∇*S*(*r*) (**G**) under tip-induced pressure (*P*_press_). (**H**) Simulated tip-induced modulation of the exciton transport ratio dominated by drift [*J*_μ_/(*J*_μ_ + *J*_D_)] and the redistributed exciton density at *r* = 0 [Δ*n*(0)] as a function of applied strain (ε).

From the experimentally observed spatial profile of the PL energy shift for X−, the strain-gradient profile as a function of the distance *r* can be derived, as shown in fig. S1. The maximum tensile strain ε is ~0.3% at the nanohole center (*r* = 0). Since the bandgap energy (*u*) and ε have given the linear relationship, with Δ*u*/Δε ≈ 100 meV %^−1^ (*30*), the bandgap-energy modification profile Δ*u*(*r*) can also be determined. Figure 4A shows this profile Δ*u*(*r*) with exhibiting Δ*u*(0) = −30 meV. Using Δ*u*(*r*), we can investigate distinct exciton behaviors in nanoscale 0D quantum wells, e.g., transport and confinement. Exciton transport within the strain-gradient structure is governed by the competition between the drift current *J*_μ_ and the diffusion current *J*_D_. *J*_μ_ and *J*_D_ are defined as μ*n*(*r*)∇*u*(*r*) and *D*∇*n*(*r*), respectively, where μ is the mobility and *D* is the diffusion coefficient. The Einstein relation, *D* = μ*k*_B_*T*, links *D* to Boltzmann’s constant *k*_B_ and temperature *T*. ∇*u*(*r*) and ∇*n*(*r*) represent spatial gradients of the bandgap energy and exciton density (*n*), respectively. In our experiment, the exciton density profile *n*(*r*) is determined by optical excitation. To quantitatively evaluate *J*_μ_ and *J*_D_, the exciton generation rate *S*(*r*) is introduced, given by $S\left(r\right)=\frac{I_{0}}{2{\pi \sigma }^{2}}e^{-r^{2}/2\sigma^{2}}$, where *I*_0_ is the amplitude of the optical intensity and $\sigma =\text{FWHM}/2\sqrt{2\text{ln}2}$ is the spatial distribution of the Gaussian beam profile. In strain-gradient structures, *J*_μ_ is expected to dominate over *J*_D_, satisfying the inequality of *D*∇*n*(*r*) + μ*n*(*r*)∇*u*(*r*) < 0. Substituting the relations of *D*/μ = *k*_B_*T* and *n*(*r*) = *S*(*r*), this inequality simplifies to *k*_B_*T* < −*S*(*r*)∇*u*(*r*)/∇*S*(*r*). This equation provides insight into the spatial separation between drift-dominant and diffusion-dominant regions, depending on the temperature. For the strain-induced MoS_2_ ML on the nanohole, we observe that exciton drift dominates across nearly the entire nanohole area (Fig. 4A, middle), even at room temperature (*k*_B_*T* = 25 meV). This dominance results in highly efficient exciton funneling and confinement at the nanohole center. To quantify *n*(*r*), we use a drift-diffusion model (*2*, *56*, *57*)

$$\nabla \left[D\nabla n\left(r\right)\right]+\nabla \left[\mu n\left(r\right)\nabla u\left(r\right)\right]-\frac{n\left(r\right)}{\tau }-n^{2}\left(r\right)R_{A}+S\left(r\right)=0$$ (1)

where τ and *R*_A_ represent the exciton lifetime and Auger recombination rate, respectively. Using material parameters τ = 10 ns, *D* = 2.1 cm^2^ s^−1^, and *R*_A_ = 3.5 cm^2^ s^−1^ from the literature (*5*, *30*), we solve for *n*(*r*), as shown in Fig. 4A (bottom), exhibiting *n*(0) = ~1.4 × 10^12^ cm^−2^. In our model, we can define the exciton confinement efficiency η_c_ as

$$\frac{\int_{0}^{r_{\mu }}n\left(r\right)\mathrm{rdr}}{\int_{0}^{r_{\text{hole}}}n\left(r\right)\mathrm{rdr}}$$ (2)

where *r*_μ_ represents the region where the *J*_μ_ dominates over the *J*_D_ (gray region in Fig. 4A), and *r*_hole_ is the nanohole radius (250 nm). We derive η_c_ = ~98%, which indicates that exciton confinement is highly effective across almost the entire nanohole. This strong confinement effect in the 0D nanohole is distinctly different from the behavior observed in 1D nanogaps, where there is no confinement along the longitudinal direction (*49*). Unlike previous studies that focused primarily on exciton funneling efficiency (*11*–*18*), our work provides the framework for quantifying confinement efficiency in nanoscale strain–engineered platforms. This metric enables direct comparison of spatial localization performance across different device geometries and provides a criterion for evaluating excitonic confinement quality. As shown in figs. S7 and S8, the maximum exciton density at the 0D nanohole center is approximately twice that of the 1D nanogap center. In addition, the nanohole achieves a remarkably small local confinement area at the nanohole center. We define this confinement region as the area where the strain-induced potential exceeds the thermal energy (*k*_B_*T*) at room temperature. On the basis of our simulation, this corresponds to a radial distance of ~214 nm from the nanohole center. This localized region provides a clear understanding of the mechanism responsible for the formation of stable X_L_. Such nanoscale control over exciton confinement provides a foundation for spatially controlled exciton harvesting applications, although our current demonstration focuses on fundamental excitonic photophysics.

We further extended our experiment using tip-induced pressure applied via an AFM tip to demonstrate the dynamic modulation of exciton confinement and X_L_ emission (see Materials and Methods for details) (*32*, *58*–*60*). For the nonannealed sample (with an H_2_O layer), gradual displacement of the AFM tip (up to a maximum of 50 nm) pressing the MoS_2_ crystal results in an ~140% increase in the PL intensity of X−, accompanied by a slight decrease in X_0_ PL intensity (Fig. 4, B and C). This behavior is attributed to the increased nanolocal strain, which reduces the bandgap energy and increases the *E*_b_. This strain-enhanced exciton and electron funneling promotes exciton-to-trion conversion, as previously described in Fig. 3. Figure S9 demonstrates that trion intensity is more sensitive to strain variations induced by tip-induced pressure, confirming the strain-dependent exciton-to-trion conversion. On the other hand, under the same experimental condition, the annealed sample (without an H_2_O layer) shows an ~120% enhancement in X_L_ PL intensity, enabling high-purity X_L_ emission. This enhancement follows a similar process but occurs without excess electrons, leading to improved X_L_ emission characteristics. Upon gradual release of the tip pressure, the emission properties are fully and reversibly restored to their original states. This reversible behavior demonstrates the potential of tip-induced pressure for dynamic control of nanoexcitonic emission in the X_L_ state. Figure S10 presents PL spectra of MoS_2_ without and under tip-induced pressure, showing an enhancement in X_L_ emission. Figure S11 presents PL peak energy shifts of X_0_, X−, and X_L_ as functions of tip-induced displacement under applied and released pressure conditions, demonstrating dynamic strain-induced bandgap modulation. Simulation results (Fig. 4, F and G) show the spatial profile of Δ*u*(*r*) and -*S*(*r*)∇*u*(*r*)/∇*S*(*r*) with respect to the tip-induced pressure (*P*_press_), reflecting the trends observed experimentally. In addition, Fig. 4H presents the simulated exciton current ratio dominated by drift [*J*_μ_/(*J*_μ_ + *J*_D_), blue] and the redistributed exciton density at *r* = 0 [Δ*n*(0), red] as functions of ε induced by the tip pressure. With a slight increase in ε of ~0.1%, *J*_μ_/(*J*_μ_ + *J*_D_) is increased by ~3%, leading to enhanced exciton confinement at the nanohole center while effectively suppressing exciton escape via diffusion. Furthermore, the drift-driven increase in Δ*n*(0) by over twofold resulted in notably enhanced X_L_ emission. In comparison, the 1D nanogap exhibits a much smaller Δ*n*(0) (gray) due to diffusion along the unconstrained axis, which prevents the realization of bright X_L_ emission at room temperature. This highlights the superior exciton confinement and X_L_ emission achieved with the nanohole geometry.

## DISCUSSION

In this study, we report bright, room-temperature X_L_ emission from strain-engineered MoS_2_ ML on metallic nanoholes. Using thermal annealing, we eliminate the interfacial H_2_O layer between MoS_2_ and the metallic substrate, thereby promoting efficient electron quenching and notably reducing trion formation, and finally leading to high population of exciton states with enhanced emission QY. These excitons then efficiently funnel into 0D quantum well at the nanohole center with increased binding energy, resulting in highly stable and deterministic X_L_ emission at room temperature. In addition, we demonstrate that tip-induced gigapascal-scale pressure provides a dynamic and reversible means of modulating exciton transport and emission, allowing precise control over X_L_ properties. By achieving room-temperature X_L_ emission and control with high-QY on a scalable platform, our work provides a previously unidentified strategy for quantum nanophotonic devices, including high-efficiency excitonic light sources, strain-tailored quantum information platforms, and highly tunable optoelectronic applications.

## MATERIALS AND METHODS

### Growth of MoS_2_ film

MoS_2_ films were synthesized using a two-zone chemical vapor deposition (CVD) system. A 0.02 M sodium molybdate aqueous solution and sulfur powder served as Mo and S precursors, respectively. The sodium molybdate precursor solution was spin-coated at 3000 rpm onto a 1 cm–by–1 cm SiO_2_/Si substrate. The CVD system, equipped with a 2-inch (5.08-cm) quartz tube, used an upstream zone for vaporizing the sulfur powder, while the downstream zone served as the growth zone. The sulfur powder and the Mo precursor–coated SiO_2_/Si substrate were placed at the centers of upstream and downstream zones, respectively. To remove residual gases, the quartz tube was purged with high-purity (99.9999%) Ar gas at a flow rate of 700 sccm for 5 min. Subsequently, the temperatures of the upstream and downstream zones were ramped to 210° and 750°C, respectively, for 10 min, and maintained for another 10 min to promote MoS_2_ film growth. After growth, the quartz tube naturally cooled down to room temperature.

### Transfer of MoS_2_ film

A dry transfer technique was used to achieve a clean interface between the MoS_2_ film and the target substrate, preventing the ML from being drawn into nanogaps or nanoholes when using the wet transfer technique. The as-grown MoS_2_ film on the SiO_2_/Si substrate was carefully submerged in deionized water, allowing it to float on the water surface. This floating MoS_2_ film was then scooped up using PDMS layer and dried in an oven at 80°C for 3 min. Subsequently, the MoS_2_ film on the PDMS was inverted and attached to nanogaps and nanohole substrate. After ramping up the temperature to 90°C, the PDMS layer was then slowly detached from the substrate.

### Fabrication of Au nanogaps using FIB milling

Au nanogaps and nanoholes were fabricated using focused ion beam (FIB) milling. A 100-nm-thick Au film was deposited on a glass substrate using an electron-beam evaporator (KVT, KVE-ENS40004) at an evaporation rate of 2 Å/s. No adhesion layer between the Au film and the substrate was used. FIB milling (FEI, Helios NanoLab G3 CX) was conducted at a fixed ion beam acceleration voltage of 30 kV, with beam currents of 1.1 and 24 pA, depending on the desired nanogaps and nanoholes dimensions.

### PL spectroscopy setup

PL measurements were conducted using a homebuilt spectroscopy setup. The sample of MoS_2_ transferred onto the Au nanogaps and nanoholes was loaded on a three-axis positioning stage (*XYZ* linear stage, M-562-XYZ, Newport) for *XY* scanning. A He-Ne laser (593.5 nm) was focused onto the sample using a microscope objective (numerical aperture = 0.8, LMPLFLN100X, Olympus). PL signals were collected in a backscattered geometry through the same objective and filtered using an edge filter (FEL0550, Thorlabs) to cut off the fundamental laser line. The PL signals were then dispersed onto a spectrometer (*f* = 320 mm, Monora320i, DXG) and imaged with a thermoelectrically cooled charge-coupled device (DU971-BV, Andor) to acquire PL spectra. The beam splitter in our polarization-resolved PL setup exhibits slight birefringence, causing polarization-dependent transmittance that varies with incident angle. To correct this, we measured the transmittance profile as a function of polarization angle under the same optical geometry and applied the resulting offset to the PL data. Before measurements, the spectrometer was calibrated using an argon-mercury lamp. All PL measurements used a 300 g/mm grating blazed to 800 nm, providing a spectral resolution of 0.31 nm.

### Tip-induced pressure engineering

For tip-induced pressure engineering, MoS_2_ ML samples on Au nanoholes were mounted on a piezoelectric transducer (P-611.3X, Physik Instrumente) for atomic force feedback, with a positioning precision of <0.1 nm. Shear-force AFM was used using an Au tip (radius of curvature: ~10 nm), fabricated through an optimized electrochemical etching process and affixed to a quartz tuning fork (resonance frequency: 32.768 kHz). Tip-sample distance was controlled via shear-force AFM using a digital AFM controller (R10, RHK Technology). Tip-induced pressure was induced by precisely positioning the Au tip above the MoS_2_ at Au nanohole and precisely adjusting the *z*-axis position.
