# Supplementary material for: Highly radiative emission of room temperature–localized excitons enabled by charge-neutralized 0D quantum wells in 2D semiconductors
Source: Sci Adv. 2026 Mar 13;12(11):eady2186. doi: 10.1126/sciadv.ady2186 (PMC12985671; doi:10.1126/sciadv.ady2186)
Supplement: Supplementary file 1 — Figs. S1 to S11 Supplementary Text Table S1 [file sciadv.ady2186_sm.pdf]

Science Advances
AAAS

Supplementary Materials for

Highly radiative emission of room temperature–localized excitons enabled by charge-neutralized 0D quantum wells in 2D semiconductors

Taeyoung Moon *et al.*

Corresponding author: Yung Doug Suh, ydsuh@unist.ac.kr; Kyoung-Duck Park, parklab@postech.ac.kr

Sci. Adv. **12**, eady2186 (2026)
DOI: 10.1126/sciadv.ady2186

This PDF file includes:

Figs. S1 to S11
Supplementary Text
Table S1

S1. Exciton-to-trion conversion in strained MoS_2 ML before thermal annealing.

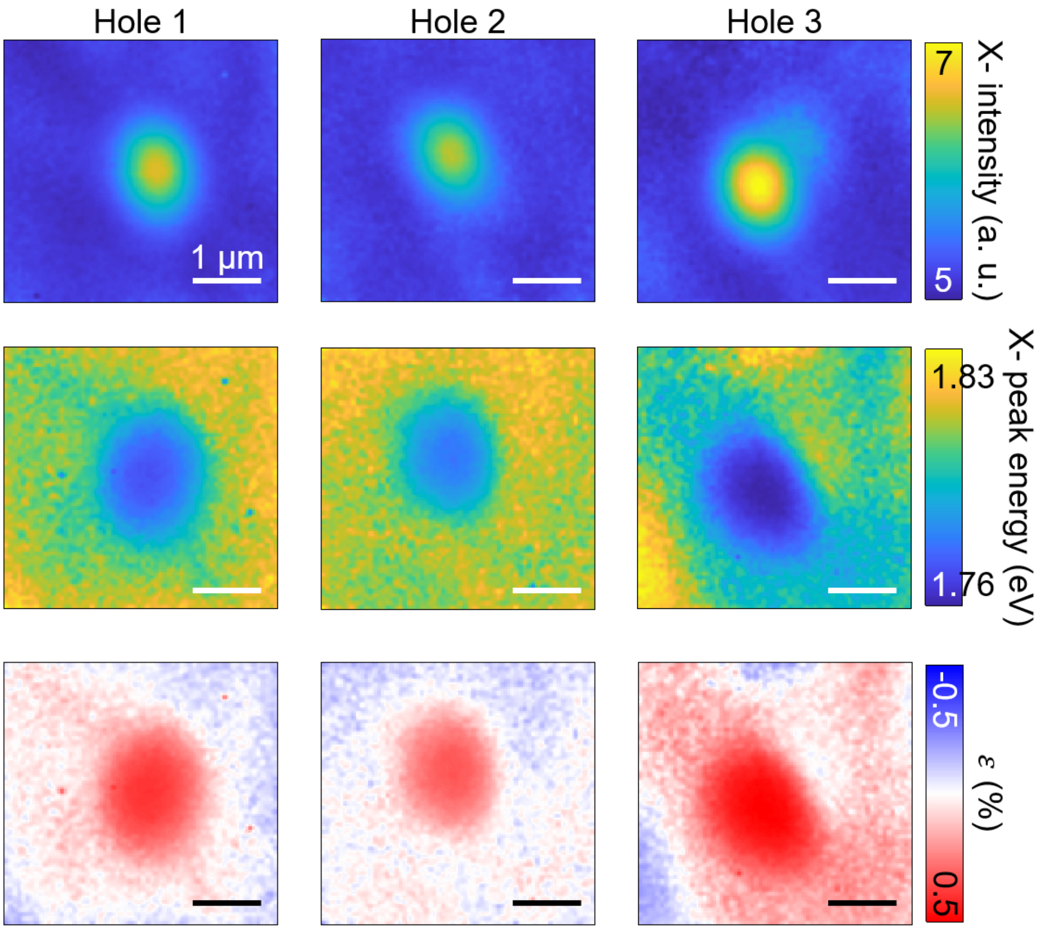

Fig. S1. Hyperspectral images for MoS_2 ML on the different 500 nm width nanohole. Top: X- PL intensity maps. Middle: X- PL peak energy maps. Bottom: strain (ε) profile.

Fig. S1 shows localized properties of PL peak energy redshift at nanohole sites, indicating well-established strain fields. This strain facilitates efficient exciton-to-trion conversion, as evidenced by the enhanced X- intensity in these regions. The bottom panel further illustrates the strain profile, where tensile strain (red) is concentrated at the nanohole center, reducing the bandgap and inducing a redshift in X- peak energy, while compressive strain (blue) at the periphery leads to a slight blueshift. The correlation between the peak energy shift and the intensity increase from Hole 1 to Hole 3 suggests a direct relationship between the strain magnitude and the efficiency of X- formation.

S2. Simulation data of X_0 and X- distribution before and after electron quenching.

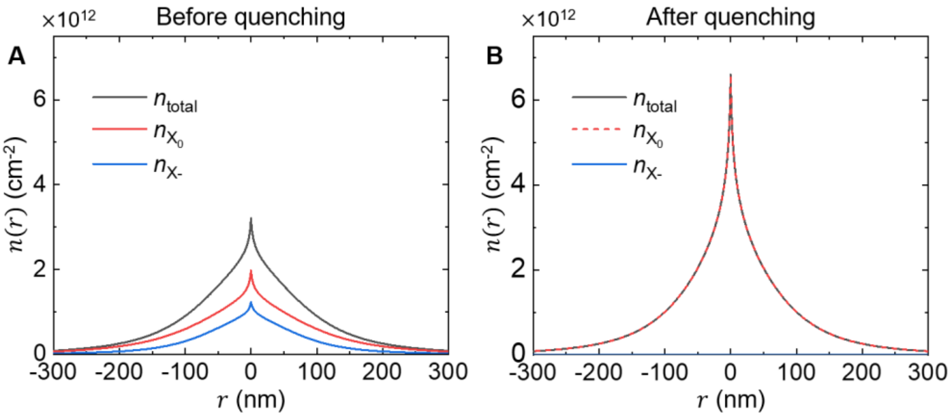

Fig. S2. Spatial distribution of X_0 and X- before and after electron quenching. (A) Distribution of total exciton, X_0, and X- before quenching. (B) The same distributions after electron quenching to the Au film.

Through the drift-diffusion equation, we calculate the spatial distribution of the X_0 and X- before and after electron quenching to the Au film. Before electron quenching (Fig. S2A), electrons in the strained MoS_2 ML facilitate X- generation via exciton-to-trion conversion, resulting in significant X- population. After electron quenching (Fig. S2B), electrons were quenched to the Au film, severely restricting X- generation. This quenching process reduces Auger recombination of excitons, enabling more efficient exciton funneling to the minimum bandgap region modulated by strain engineering. Consequently, the dominant X_L emission observed in the strained MoS_2 ML can be attributed to exciton funneling into the localized exciton state after electron quenching.

S3. X_L distribution at the strained MoS_2 ML after thermal annealing.

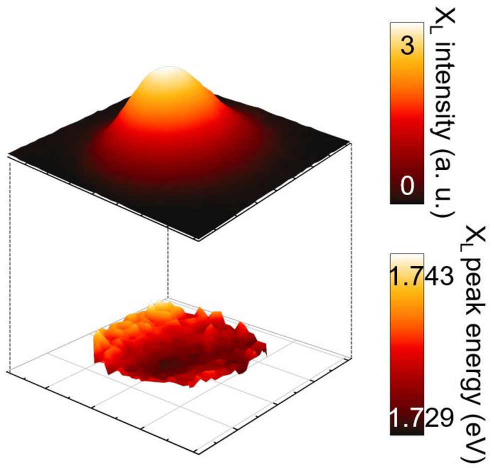

Fig. S3. Hyperspectral imaging of X_L PL intensity and peak energy at the strained MoS_2 ML. The intensity peak and energy dip of X_L confirm strain-induced exciton confinement.

S4. PL Characterization of X_L at Cryogenic Temperature (5 K)

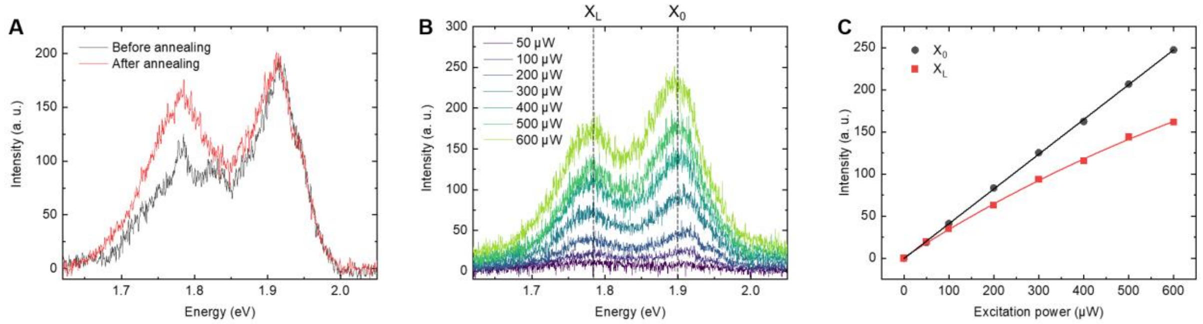

Fig. S4. PL spectra at cryogenic temperature (5 K) on the nanohole center. (A) PL spectra before (black) and after (red) thermal annealing. (B) Excitation power dependent PL spectra (50–600 μW), with the X_0 and X_L peaks indicated by dashed lines. (C) PL intensities of X_0 and X_L as a function of excitation power.

We performed low-temperature PL measurements to investigate the properties of X_L and their response to thermal annealing. Before annealing, the spectrum was dominated by X_0 with several sharp features corresponding to X_L emission. Thermal annealing suppressed non-radiative decay channels and reduced X- formation, significantly enhancing PL QY and revealing multiple previously dark localized states that appeared as a broad X_L peak shown in Fig. S4A. The persistent X_0 emission at 5 K is attributed primarily to far-field collection from unstrained regions beyond the nanohole. In addition, a thermal barrier between X_0 and X_L states cannot be entirely excluded. Thus, high spatial resolution near-field spectroscopy can resolve the potential role of barrier dynamics in future studies.

Power-dependent measurements confirmed the excitonic nature of this emission. While X_0 PL intensity scales linearly with excitation power, X_L exhibits sublinear saturation behavior, providing definitive evidence of quantum confinement and finite density of localized states (Fig. S4B–C). This saturation is characteristic of discrete excitonic levels within strain-induced potential wells. The number and energy distribution of localized states are governed by the strain potential profile, which can be controlled through nanohole geometry. Our 500 nm diameter was designed to balance efficient exciton collection with adequate MoS_2 suspension to avoid edge effects, resulting in a multilevel potential that supports several excitonic states (*9, 12, 56, 57*). This explains both the observed spectral broadening after thermal annealing and the incomplete saturation at room temperature, where thermal redistribution ($k_BT \approx 25$ meV) and phonon-induced non-radiative pathways prevent complete state filling.

S5. PL spectra for linearly polarized X_L emission from strained MoS_2 ML

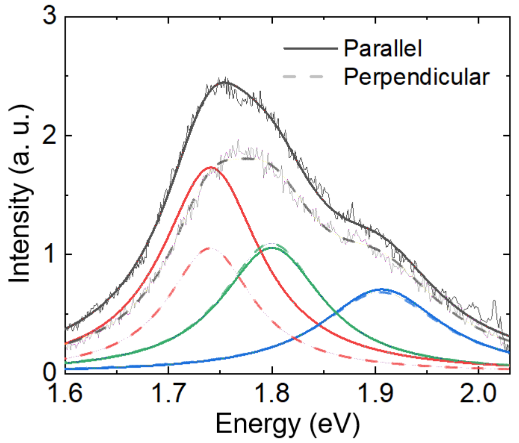

Fig. S5. Comparison of PL spectra under different polarization conditions in strained MoS_2 ML.

Fig. S5 presents the PL spectra measured under two different polarization conditions to highlight the linearly polarized emission of X_L in strained MoS_2 ML. The solid lines represent the PL spectra measured along the polarization direction aligned with the emission of X_L, while the dashed lines correspond to the spectra obtained in the perpendicular polarization to this direction. The black line represents the fitted spectra, while the blue, green, and red lines correspond to the fitted components for X_0, X-, and X_L, respectively. The black dots indicate the raw data points.

S6. Polarization dependence of X_0 and X_L at the various nanoholes.

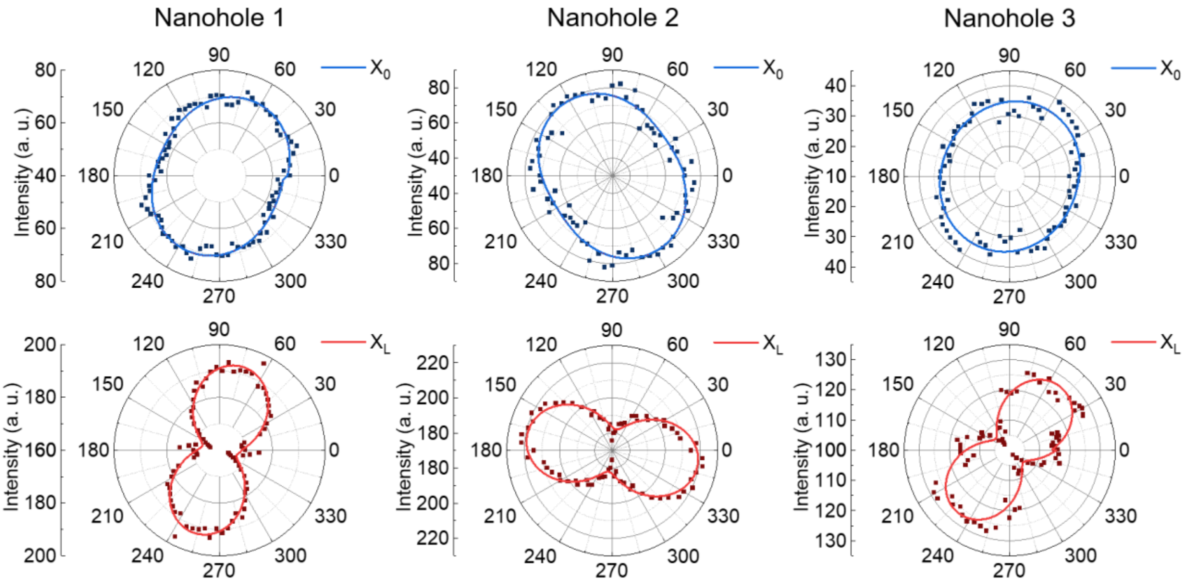

Fig. S6. Polarization-resolved PL of X_0 (top row) and X_L (bottom row) at three independent nanoholes.

The polar intensity maps were corrected for polarization-dependent instrumental losses to reveal intrinsic emission properties. In every case, X_0 shows nearly isotropic emission, while X_L exhibits a pronounced two-lobed dipole pattern. The reproducible dipolar signature of X_L across multiple holes confirms that this strong linear polarization arises from the strain-induced, spatially confined excitonic states at the nanoholes. This systematic polarization selectivity where only X_L shows strong dipolar emission while X_0 remains unpolarized, demonstrates that these effects are intrinsic to the localized exciton states rather than experimental artifacts. The corrected intensity scaling properly represents the perpendicular-to-parallel intensity ratio, addressing the apparent inconsistency between the polar plots and raw spectral measurements shown in Fig. S5.

S7. Redistributed exciton density via modified bandgap at the nanogap and nanohole.

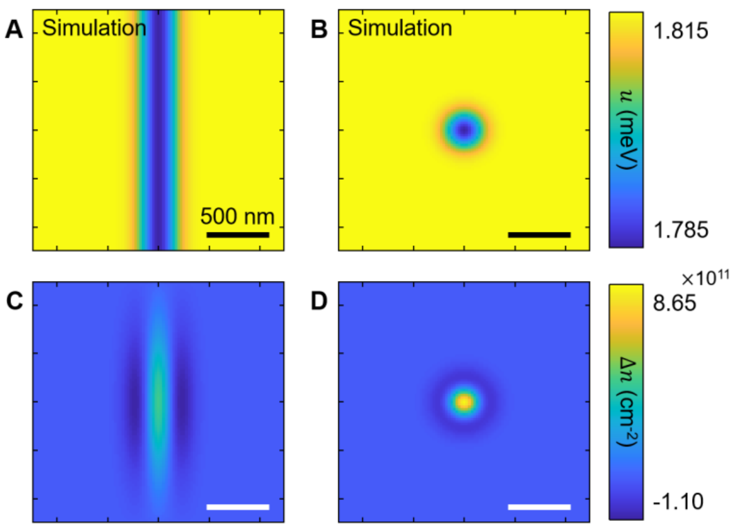

Fig. S7. Quantified exciton density within modulated bandgap regimes at nanogap and nanohole. Simulation results of the modified bandgap (u) profiles of MoS_2 at the nanogap (A) and nanohole (B). Redistributed exciton densities at the nanogap (C) and nanohole (D) induced by the strain gradient.

The induced strain at the nanogap and nanohole modifies the bandgap of MoS_2. These modified bandgap profiles, exhibiting the same bandgap shift in both structures, were analyzed using the drift-diffusion equation, as detailed in the main text. Fig. S7A-B show the modified bandgap profiles, where localized minima are observed at the centers of the nanogap and nanohole, respectively. Fig. S7C-D show the redistribution of exciton densities due to strain gradients. The nanohole exhibits more effective exciton localization than the nanogap, owing to its intrinsic two-dimensional potential confinement. This distinctive structural advantage of nanohole ensures efficient exciton trapping at the lowest bandgap minima, which is essential for realizing high X_L emission. These findings highlight the robust excitonic confinement of nanohole, enhancing its potential as a key platform for developing X_L emitters and optoelectronic technologies.

S8. Comparison of redistributed exciton density at the nanohole and nanogap.

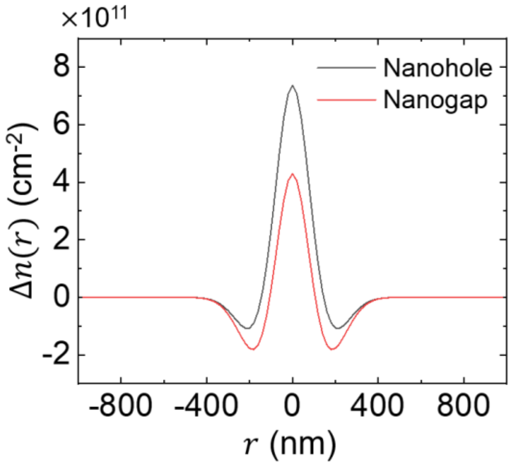

Fig. S8. Line profile of redistributed exciton density at the nanohole and nanogap.

Fig. S8 presents the simulation of redistributed exciton densities at the nanohole and nanogap induced by strain. The nanohole demonstrates an exciton density ~2 times larger than that of the nanogap at the lowest bandgap region, emphasizing its enhanced capability for exciton localization.

S9. Spatial distribution of X_0 and X- before and after additional strain engineering.

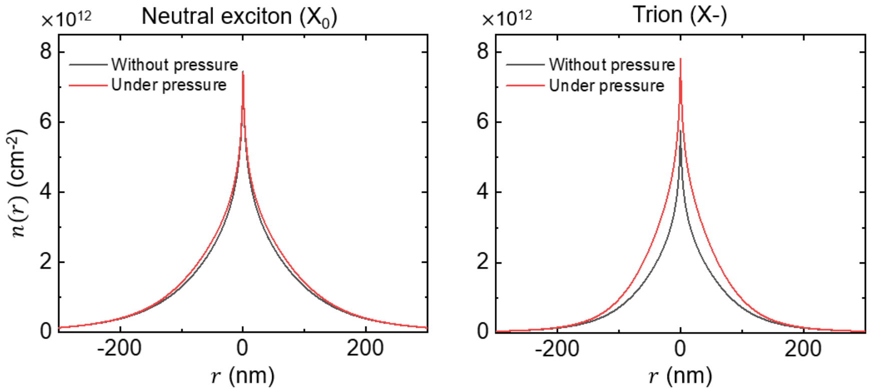

Fig. S9. Spatial modulation of X_0 and X- excitonic states under strain engineering. Spatial distribution of X_0 and X- in a MoS_2 ML without pressure (black line) and under pressure (red line). Left: X_0 distribution shows increased density at the center after pressure application. Right: X- distribution exhibits a more significant increase in density and broader spatial distribution under pressure.

We calculate the spatial distribution of X_0 and X- in response to tip-induced pressure via AFM tip. Through tip-induced pressure engineering, the X_0 intensity at the nanohole slightly increased compared with the condition without pressure. In contrast, the X- intensity at the nanohole significantly increased compared to X_0. Therefore, this result explains the significant increase in X- and X_L intensities observed in Fig. 4C-E, which exceeds the increase of X_0

S10. Tip-induced pressure at the strained MoS_2 ML after thermal annealing.

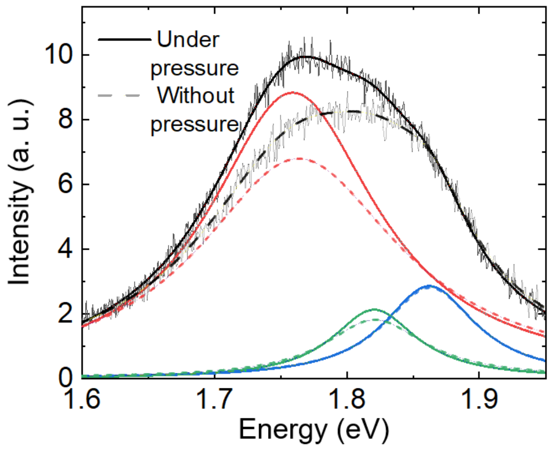

Fig. S10. PL spectra of MoS_2 ML without and under tip-induced pressure. Dashed lines represent the spectra without tip-induced pressure, while solid lines show the spectra under tip-induced pressure. The graph demonstrates a significant increase in PL intensity and spectral shifts, indicating enhanced exciton localization and strain-induced bandgap modulation. The black line represents the fitted spectra, while the blue, green, and red lines correspond to the fitted components for X_0, X-, and X_L, respectively. The black dots indicate the raw data points.

S11. Dynamic modulation of X_0, X-, and X_L PL peak energy via tip-Induced pressure.

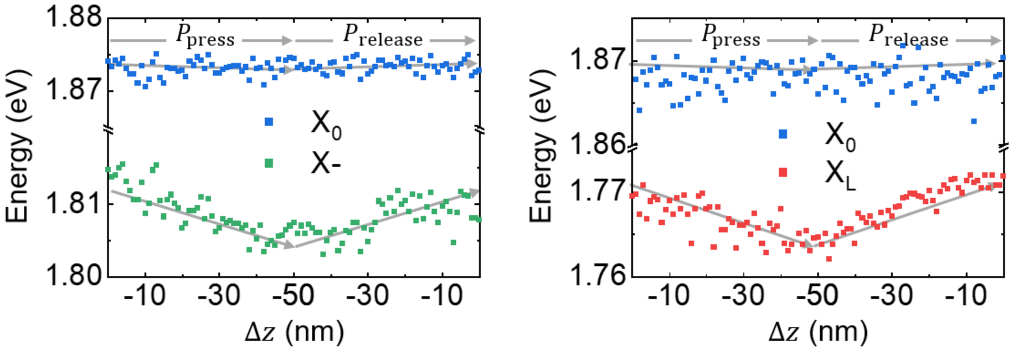

Fig. S11. PL peak energy shifts of X_0, X-, and X_L as a function of tip-induced displacement Δz under applied pressure (P_{press}) and release (P_{release}) conditions. Left panel: The PL peak energy of X_0 and X−, before thermal annealing. Right panel: PL peak energy of X_0 and X_L after thermal annealing. The results demonstrate that tip-induced pressure dynamically modifies the bandgap of MoS_2, enabling precise modulation of excitonic properties.

S12. Quantification of PL Quantum Yield Enhancement by Thermal Annealing.

To quantify how thermal annealing affects the PL QY of MoS_2 ML on the nanohole and nanogap structures and Au substrate, we apply rate-equation model of (*32*), which enable calculating PL QY in terms of the trion-to-exciton intensity ratio. The model considers the competition between radiative and nonradiative recombination processes for both X_0 and X-. Denoting by I_{X_0} and I_{X-} the integrated PL peak areas of the X_0 and X-, and by $\tau_{X_0,r}$, $\tau_{X-,r}$, $\tau_{X_0,non-r}$, and $\tau_{X-,non-r}$ their radiative and nonradiative lifetimes, respectively, one obtains the PL QY as:

$$\mathrm{QY} = \frac{\frac{n_{X_0}}{\tau_{X_0,r}} + \frac{n_{X-}}{\tau_{X-,r}}}{\frac{n_{X_0}}{\tau_{X_0,r}} + \frac{n_{X-}}{\tau_{X-,r}} + \frac{n_{X_0}}{\tau_{X_0,non-r}} + \frac{n_{X-}}{\tau_{X-,non-r}}} = \frac{\frac{1}{\tau_{X_0,r}} + \frac{R}{\tau_{X-,r}}}{\frac{1}{\tau_{X_0,r}} + \frac{1}{\tau_{X_0,non-r}} + R(\frac{1}{\tau_{X-,r}} + \frac{1}{\tau_{X-,non-r}})},$$

where the population ratio

$$R = \frac{n_{X-}}{n_{X_0}} = \frac{\tau_{X-,r} I_{X-}}{\tau_{X_0,r} I_{X_0}}.$$

To parameterize our rate-equation model, we initially adopted the lifetimes reported in literature (*32*): $\tau_{X_0,r}$ = 8 ns, $\tau_{X-,r}$ = 110 ns and $\tau_{X-,non-r}$ = 0.05 ns, to evaluate the PL QY using the rate-equation model. The $\tau_{X_0,non-r}$ was omitted from the calculation due to its much longer timescale, simplifying the rate equation.

Table S1. Trion-to-exciton intensity ratio I_{X-}/I_{X_0}, derived population ratio R, and resulting total PL QY before and after thermal annealing.

Structure & Condition	I_{X-}/I_{X_0}	R	PL QY (%)	Enhancement Factor (After / Before)
Fig. 2**C** (substrate, before annealing)	0.25 ± 0.004	3.4 ± 0.055	0.23 ± 0.003%	
Fig. 2**D** (substrate, after annealing)	0.12 ± 0.0006	1.6 ± 0.008	0.42 ± 0.001%	~1.8
Fig. 2**E** (nanogap, before annealing)	0.96 ± 0.015	13 ± 0.21	0.093 ± 0.001%	
Fig. 2**F** (nanogap, after annealing)	0.17 ± 0.0007	2.3 ± 0.01	0.31 ± 0.001%	~3.3
Fig. 3**D** (nanohole, before annealing)	1.5 ± 0.036	20 ± 0.50	0.076 ± 0.002%	
Fig. 3**E** (nanohole, after annealing)	0.0041 ± 0.0005	0.055 ± 0.007	10 ± 1.3%	~130

Thermal annealing systematically reduces the trion-to-exciton population ratio R, thereby significantly enhancing the total PL QY across all nano-platforms tested. The kinetic model considers X_0 and X- dynamics. Since X_L states are neutral excitons localized by strain, the model predictions directly apply. At the nanohole center, X_L emission dominates (Fig. S1) as excitons funnel into strain-localized states. The calculated ~130× QY enhancement thus reflects X_L emission. In pristine monolayer MoS_2, the PL QY typically remains below ~1%, and even in structures engineered for exciton funneling, the QY is often too low to resolve X_L emission at room temperature. In contrast, our thermal annealing approach not only

suppresses nonradiative recombination pathways but also enhances exciton confinement, collectively enabling bright, room-temperature X_L emission that was previously unobservable.

The enhancement in PL QY varies depending on the nanostructure geometry, with approximately 3.3-fold improvement in nanogap and up to 130-fold in nanohole. This pronounced difference arises from the efficiency of charge extraction. In nanohole, the radially symmetric geometry allows electrons to be quenched into the metal substrate from all directions, effectively removing excess charges. In contrast, nanogap permits charge extraction only along two lateral directions, making the process less efficient. As a result, nanohole exhibits significant reduced trion formation and nonradiative decay, leading to enhanced radiative X_L emission at room temperature.
